# The effects of an abrupt increase in taxes on candy and soda in Norway: an observational study of retail sales

**DOI:** 10.1186/s12966-020-01017-3

**Published:** 2020-09-14

**Authors:** Bente Øvrebø, Torleif B. Halkjelsvik, Jørgen R. Meisfjord, Elling Bere, Rannveig K. Hart

**Affiliations:** 1 Faculty of Health and Sport Sciences, University of Agder, Kristiansand, Norway; 2 Centre for Evaluation of Public Health Measures, Norwegian Institute of Public Health, Oslo, Norway; 3 Department of Health and Inequalities, Norwegian Institute of Public Health, Oslo, Norway; 4 Department of Alcohol, Tobacco and Drugs, Norwegian Institute of Public Health, Oslo, Norway

**Keywords:** Public health tax, Sugary drink tax, SSB-tax, Retail sales, Sin tax, Quasi-experiment

## Abstract

**Background:**

Fiscal policies are used to promote a healthier diet; however, there is still a call for real-world evaluations of taxes on unhealthy foods and beverages. We aimed to evaluate the effect of an abrupt increase, of respectively 80 and 40%, in the excising Norwegian taxes on candy and beverages on volume sales of candy and soda. We expected sales to fall.

**Methods:**

We analyzed electronic point of sale data covering approximately 98% of volume sales of grocery stores in Norway. In two pre-registered models with weekly (log-)sales of taxed candy and soda from 3884 individual stores, we modeled the difference between the jump (discontinuity) in the trend around the time of the increase in taxes and the corresponding jump in the trend in a control season from the previous years (Model 1). In addition, we modeled the difference between the intervention and the control season in their changes in average sales (Model 2).

**Results:**

Model 1 showed a 6.1% (one-sided 95% CI: not applicable (NA), 23.4, *p*-value = 0.26) increase and a − 3.9% (95% CI: NA, 4.9, p-value = 0.23) reduction in the differences in the jump in the trends, for candy and soda, respectively. The second model showed a relative decrease of − 4.9% (95% CI: NA, 1.0, *p*-value = 0.08) in the average sales of candy and an increase of 1.5% (95% CI: NA, 5.0, p-value = 0.24) in sales of soda. Supplementary analyses suggested that the results were sensitive to clustering on the time dimension.

**Conclusions:**

When using two different quasi-experimental designs to model changes in volume sales of taxed candy and soda, we were not able to detect reductions in sales that coincided with an increase in the taxes. Variation across time makes it difficult to detect potentially small changes in sales even when using an entire country’s worth of sales data on the level of individual stores. We speculate that the tax increases were too modest to affect the prices to alter sales sufficiently.

## Background

The prevalence of overweight and obesity is a burden worldwide [1]. Overweight and obesity are associated with excessive intake of energy-dense and nutrient-poor foods [2], which may lead to increased risk of morbidity and mortality and other negative consequences affecting the individual and society in general (e.g., financial strain) [3–5]. To promote a healthier diet and tackle obesity and non-communicable diseases, the World Health Organization recommends the use of fiscal policies [6].

Historically, taxes on unhealthy products have been motivated by revenues, but lately, several countries have implemented taxes that aim to shift consumer consumption towards a healthier diet [7], with taxes on sugar-sweetened beverages (SSBs) being the most common.

A systematic review and meta-analysis evaluating the prospective impact of food pricing on dietary consumption supports taxation as a method to reduce the intake of unhealthy foods and beverages [8]. Furthermore, taxation of SSBs has been reported to lower sales of SSBs with the potential to reduce energy and sugar intake [9]. However, a large proportion of studies on the effect of taxes on unhealthy foods and beverages are modeling or simulation studies, few are based on real-world evaluations [9, 10]. Nonetheless, a recent systematic review and meta-analysis using only real-world evaluations of SSB-taxes reported an average decline in unhealthy beverage purchases and dietary intake of 10% with a SSB-tax of 10% [11]. However, the results were strongly heterogeneous across study contexts and tax designs.

Compared to taxes on beverages, fewer countries have implemented and evaluated taxes on unhealthy foods. Mexico reported a 5.3% reduction in purchases of taxed foods after implementing an 8% tax on energy-dense nutrient-poor foods [12], whereas Hungary with a public health tax [13] and Denmark with a tax on saturated fat have reported smaller effects [14]. There is still a need for real-world evaluations of taxes on foods and beverages, especially taxes on unhealthy foods, to understand their effects in different contexts [10, 11, 15].

In November 2017, Norwegian budget negotiations led to an abrupt 80% increase in the tax on chocolate and sugar products, from 20.19 NOK (2.09 EUR) per kg to 36.92 NOK (3.82 EUR) per kg; and a 40% increase in the tax on non-alcoholic beverages, from 3.34 NOK (0.35 EUR) per liter to 4.75 NOK (0.49 EUR) per liter [16]. Both increases were implemented on January 1st, 2018. Although the taxes were increased mainly to create revenues, the potential public health benefits were emphasized by the government. With the abrupt increase in the taxes, a natural experiment setting emerged.

This study aimed to identify the effect of the abrupt increases in the existing Norwegian taxes on chocolate and sugar products and non-alcoholic beverages on the sales of these products. We expected the sales of taxed products to fall; thus, our hypotheses were directional.

## Methods

### Study design

We evaluated the potential changes in sales during an intervention season, representing the periods before and after the abrupt increase in taxes on January 1st, 2018 (weeks 30–52 in 2017 and 1–23 in 2018), against the changes in sales during a control season (weeks 30–52 in 2016 and 1–23 in 2017). To avoid the high variability in sales during Christmas and the subsequent weeks, we excluded four weeks on each side of the cutoff (January 1st) in each season. The weeks included in the main analysis are presented in Figure S1 (see Supplementary Figure S1, Additional file 1). The outcome variables were weekly volume sales of candy and soda covered by the taxes. The effects were estimated by two types of quasi-experimental designs. In Model 1 (difference-in-discontinuity), the (geometric) average sales over time were modeled flexibly with splines before and after January 1st (excluding the window of eight weeks) and allowed for different slopes in the control and intervention seasons. The effect estimate in Model 1 represents the difference between the two seasons in the breaks (jumps) of their trends before and after the time of the intervention. In Model 2, time was modeled as fixed effects per week number across the two seasons, and the effect estimate represents the difference between seasons in changes of the (geometric) average sales from the period before to after January 1st.

### Data and setting

We used longitudinal retail data, consisting of grocery stores sales data as registered at checkout scanners in the period June 2016 to June 2018 from the four largest chains in Norway, collected by the Nielsen Company Norway. Data consisted of sales in value (NOK) and volume, aggregated by product category, store, and week. When compared against the official retail sales from Statistics Norway [17], the total data set covers about 98% of the annual sales in Norwegian grocery stores [18]. This is an approximate estimate of the proportion of sales, as definitions of a grocery store may vary.

The taxes do not differentiate between sugar or artificial sweetener content. Thus, irrespective of the type of sweetener, we formed two groups of taxed products that served as our primary outcomes: candy and soda. We excluded seasonal products, products that are not typically associated with candy (marzipan, energy tablets, etc.), and bulk candy (not provided in volume sales). The taxed candy product group consisted of the following subcategories: pastilles, other sugary products, bubblegum, sweets, caramels, chocolate (bars, figures, boxes, etc.) and licorice. In the soda product category, we included all subcategories of prepared soda with added sugars and artificial sweeteners. For each grocery store, we summed up the weekly volume sales within each of the two product groups, candy in kg and soda in liter, and used the natural log of these sums in the analyses. Thus, the analyses were based on the aggregated volume sales of various product groups, not their nutritional content (for which data was not available).

### Statistical methods

The analyses were conducted in Stata version 15.1 software (StataCorp. 2017. *Stata Statistical Software: Release 15.* College Station, TX: StataCorp LLC). Details on the data preparation and statistical models are described in the pre-registered analysis plan available online (https://osf.io/pz4eg/) [19]. For each of the product groups (candy and soda), we ran two different models: Model 1 with splines and Model 2 with week number as fixed effects. Model 1 was an ordinary least squares regression with sales in log-volume as the outcome:

$$ \ln {(y)}_{i,t}={a}_{shop}+{\beta}_1\left({X}_{season,i,t}\ast {X}_{dur\ge 0i,t}\right)+{\beta}_2{X}_{season,i,t}+{\beta}_3{X}_{dur\ge 0i,t}+{\beta}_4{X}_{contr}+ yf\left({X}_{dur,i,t}\right)+\delta f\left({X}_{dur,i,t}\ast {X}_{season,i,t}\right)+{e}_{i,t}. $$

*β*_1_ is the parameter of interest (tax effect) and captures the difference between the discontinuity in the intervention and control season, comparing the jump from late November to early February (a local effect). *β*_2_ captures differences in the level between intervention and control season. *β*_3_ captures the shared jump from late November to early February across the intervention and control season. *β*_4_ is a vector of controls, including dummies for Halloween and Easter, and the value of sales of non-edible products (an exogenous proxy for total sale). *a*_*shop*_ captures fixed effects at the shop level. We modeled time trends by restricted cubic splines using the *mkspline* function in Stata with a total of three knots; one before the cutoff (week number 30), one at the cutoff (week number 5) and one after the cutoff (week number 23). *y* captures shared time trends (the splines), and *δ* captures how the intervention season deviates from this trend.

Model 2 modeled time by fixed effects of week number:

$$ \ln {(y)}_{i,t}={a}_{week}+{a}_{shop}+{\beta}_1\left({X}_{season,i,t}\ast {X}_{dur\ge 0i,t}\right)+{\beta}_2\left({X}_{season,i,t}\right)+{\beta}_4{X}_{contr}+{e}_{i,t} $$

Fixed effects at the week number level (*a*_*week*_) replaced joint and separate trend modeling. We controlled for Easter and a proxy of total sales (*β*_4_), as described above. Halloween fell on the same week number and was thus not included because Model 2 includes fixed effects of week number. As there are only two seasons, the model resembles an interrupted time series design (the season dummy captures the linear time trend), but it is parameterized as a difference-in-difference model. While Model 1 captured the local change around the cutoff, accounting for trends within seasons, Model 2 gave the difference between the intervention and control season in their average change from before to after the cutoff.

To account for dependencies (e.g., autocorrelation) within geography and time, respectively, we estimated robust standard errors with two-way clustering on time and at the level of municipalities using the Stata user-written function *reghdfe* [20]. As we were only interested in the potential fall in sales, we report one-sided 95% CIs.

We ran several sensitivity tests, as described in the results section. Further, in the descriptive analyses of changes in price, we calculated the price per volume for each subcategory within the two product groups and reported the means of these subcategories for taxed candy and soda, respectively.

### Ethics

The study does not qualify as human participant research or medical research. No ethical approval was needed according to national legislation.

## Results

**Fig. 1:**
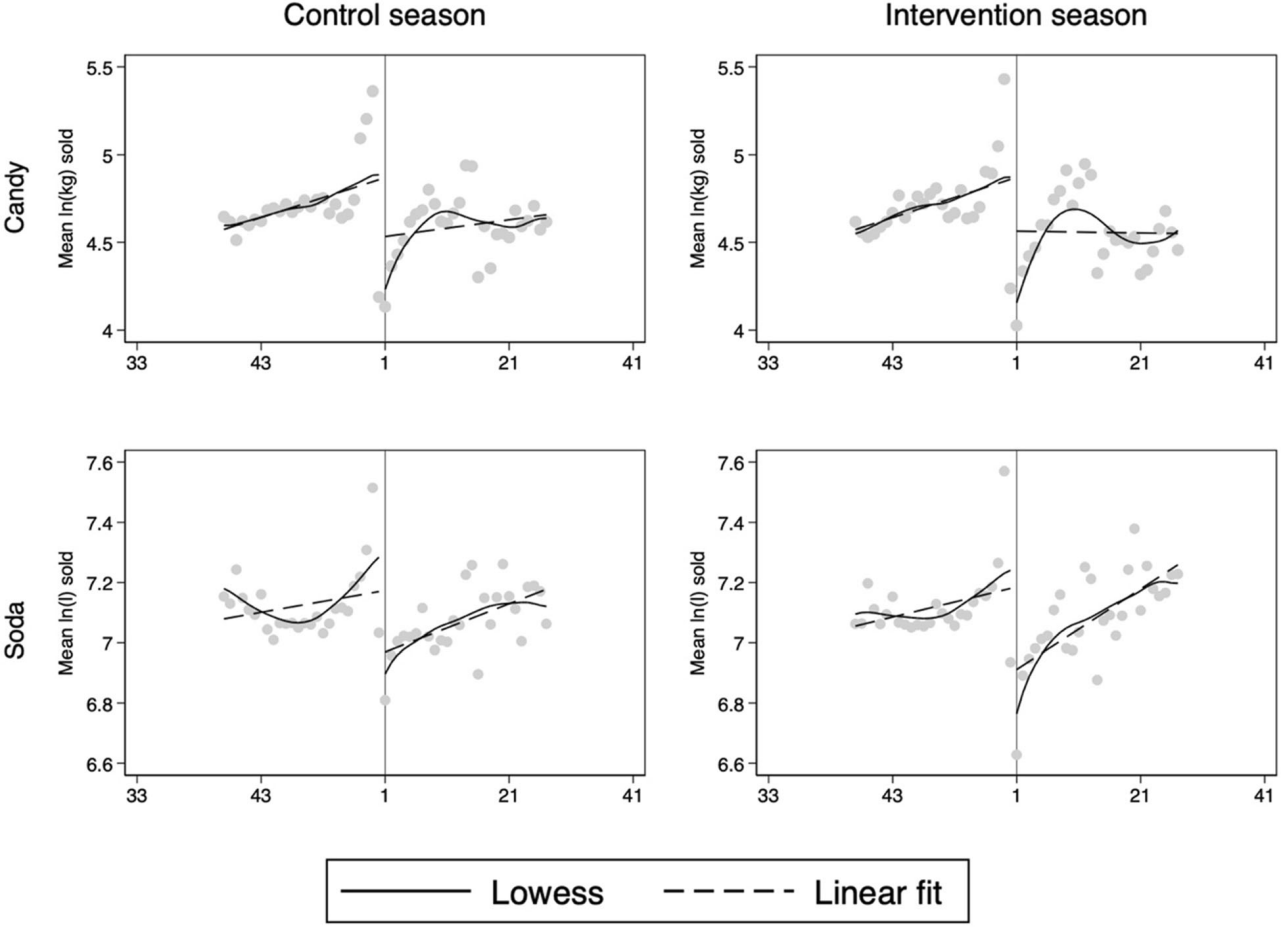
Mean ln (volume) weekly sales of taxed candy (top) and taxed soda (bottom). X-axis show week number of the year. Lowess and linear fits are for illustrative purposes

Sales data from 3884 stores were used in the analysis. Descriptive mean weekly sales of taxed candy and soda in the control and intervention season are presented in Fig. 1. The figure indicates similar trends in sales with an increase in sales in the weeks before week 52 and with a drop in sales from week 1. After week 1, the sales increase in both product groups.

Descriptive results of weekly volume sales for both product groups by each season are presented in Table 1. The upper panel shows the average sales per week before and after January 1st for the full seasons (week 27 to 52 and week 1 to 26 the following year), and the lower panel shows the average weekly sales for the seasons used in the main analyses (excluding seven weeks during the summer and excluding an eight-week window centered on January 1st).

**Table 1:** Descriptive mean (±SD) weekly store volume sales of taxed candy and taxed soda

	Taxed candy (kg)		Taxed soda (liter)	
	Control	Intervention	Control	Intervention
Complete seasons				
Pre	165 (62)	162 (52)	1882 (333)	1864 (370)
Post	143 (31)	140 (39)	1790 (210)	1812 (266)
Change	- 21 (−13.0%)	- 22 (−13.7%)	- 92 (−4.9%)	- 52 (−2.8%)
Seasons as in analyses				
Pre	148 (11)	152 (14)	1778 (116)	1788 (152)
Post	150 (31)	148 (42)	1818 (210)	1873 (238)
Change	2 (1.1%)	- 4 (−2.6%)	40 (2.3%)	85 (4.8%)

Pre/post signifies pre or post the cutoff (January 1st). *SD* Standard deviation

**Fig. 2:**
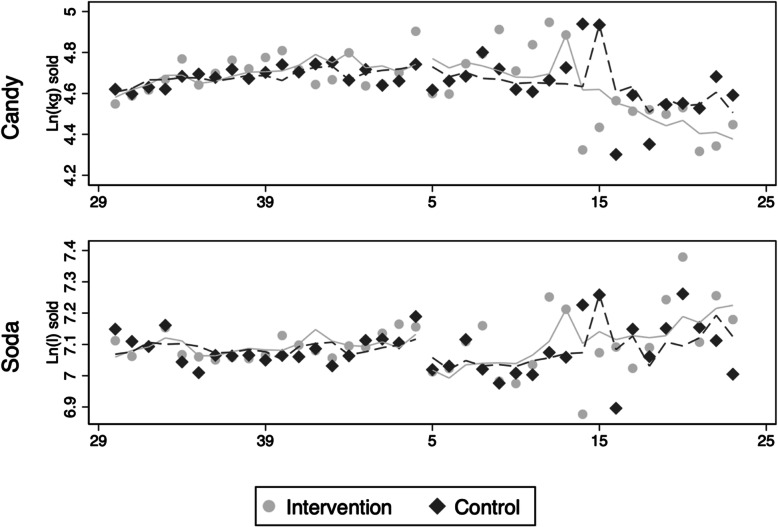
Sales of taxed candy (top) and taxed soda (bottom). Figures show intervention season (light grey line = predicted values) and control season (dashed dark line = predicted values) from Model 1. Dots represent weekly mean observations. X-axis show week number of the year

Model 1 (estimating the local effect) yielded a 6.1% increased volume sale of taxed candy in the intervention season compared to the control season, and a reduction in sales of soda corresponding to a difference of − 3.9% (Table 2). These numbers represent the differences in the estimated jumps in the trends, as illustrated in Fig. 2. As one coefficient was in the opposite direction of our predictions, and the other yielded a one-sided *p*-value of 0.23, the results were inconclusive.

**Table 2:** Exponentiated regression coefficients of the tax effect for the main models

	Candy		Soda	
	Model 1 (local)	Model 2 (average)	Model 1 (local)	Model 2 (average)
Tax effect	1.061	0.951	0.961	1.015
One-sided 95% CI	[NA, 1.234]	[NA, 1.010]	[NA, 1.049]	[NA, 1.050]
One-tailed *p*-value	0.26	0.08	0.23	0.24

*CI* Confidence intervals. *NA* Not applicable due to one-sided CIs

Model 2 estimated the difference in changes between the average sales before and after the cutoff (rather than the local jump around the cutoff). Analysis with Model 2 revealed a reduction in sales of candy corresponding to a difference of − 4.9% when comparing the intervention season to the control season (one-sided *p*-value = 0.08). The analysis of soda yielded a 1.5% increase in sales, contrary to our prediction (see Table 2). The coefficients of the two analyses using Model 2 were in the opposite direction of the local model (Model 1). A table that includes all regression coefficients in the main analyses is presented in Supplementary Table S2 in Additional file 1.

Due to the null results of our main analyses, we did not analyze potential substitute products and we did not emphasize one model over the other, as described in the preregistration [19].

### Supplementary analyses

As it is likely that the effect of the taxes varies with cross-border shopping possibilities, we conducted a subsample analysis excluding municipalities categorized as high cross-border shopping municipalities (see Supplementary Text S3, Additional file 1). Results were not different from the main analyses (Supplementary Text S4 and Table S5, Additional file 1).

To evaluate robustness in terms of the choice of periods, we excluded an additional two weeks on each side of the cutoff in each season, resulting in the exclusion of twelve weeks per season. Analyses of these data gave similar results as the main analyses, except in Model 1 for taxed candy, which showed an estimated local increase in sales of 16% in the intervention season (see Supplementary Table S6, Additional file 1).

In addition to the above pre-registered sensitivity analyses, we ran analyses based on Model 2, where the changes in the intervention season were compared against the average changes of all previous seasons for which we were able to obtain reliable data (2012–2017, five seasons). The analysis yielded coefficients corresponding to a − 3.9% one-sided 95% CI [NA, − 0.1] reduction in sales of candy (one-sided *p*-value = 0.05) and a 5.5% [NA, 9.3] increase in soda sales, one-sided p-value = 1.0, see Supplementary Table S7, Additional file 1.

Factors that change sales differentially in the control and treatment seasons (e.g., if the weather was warmer in the second part of the control season) could confound our results. Such changes would also affect product groups similar to the taxed products and can be netted out in a triple difference (DiDiD) design. To estimate DiDiD models, we included observations of sales of similar, non-taxed products into our data sets, comparing candy to snacks and soda to bottled water. The DiDiD effect estimate from this analysis shows how a taxed product group deviates from a control product group in terms of differences between the pre-post change in the intervention season and the pre-post change in the control season. These analyses showed an estimated − 7.2% [NA, 0.5] (*p*-value = 0.06) reduction for candy and 0.8% [NA, 17.3] (p-value = 0.47) increase for soda (See Supplementary Table S8, Additional file 1).

To explore how clustering on time influenced the uncertainty of the estimates in the main models, we inspected CIs and *p*-values based on standard errors that only accounted for clustering on municipalities (not on time as in the main analyses). This reduced the uncertainty substantially (e.g., Model 2 for candy gave 0.951 [NA, 0.959], p-value < 0.001), and suggests that the high level of uncertainty in the estimates is mainly driven by dependencies within time (e.g., co-movements in sales across the stores).

### Impact on prices

When hypothesizing a fall in sales, we presumed that the prices on taxed products would be higher after the intervention (the tax increases). The volume price of taxed candy increased 5.8 percentage points more in the intervention season in comparison to the control season, and the same figure for taxed soda was 8.0 percentage points (see Table 3).

**Table 3:** Mean (±SD) weekly volume price of taxed products with the season as in analysis (NOK)

	Taxed candy (price per kg)		Taxed soda (price per liter)	
	Control	Intervention	Control	Intervention
Pre	289.7 (3.8)	298.2 (6.2)	22.4 (0.4)	22.6 (0.6)
Post	296.4 (4.6)	322.3 (4.1)	21.7 (1.0)	23.7 (0.9)
Change	6.7 (2.3%)	24.1 (8.1%)	−0.7 (−3.1%)	1.1 (4.9%)

Pre/post signifies pre or post the cutoff (January 1st). *NOK* Norwegian currency (kroner). *SD* Standard deviation

## Discussion

In the context of an abrupt increase in taxes on candy and soda implemented January 1st, 2018, in Norway, we assessed the differences between the season of the intervention and a control season in terms of changes in sales of taxed products from the periods before and after January 1st. Using two different quasi-experimental models to analyze changes in volume sales, we were not able to consistently detect reductions that coincided with the increases in the taxes. The uncertainty of the effect estimates was high, which can be attributed to high variation in sales over time (that is not captured by modeling of covariates, seasonality, and trends). There was no reliable local effect before to after the intervention, and no decline in the average sales of soda, but the reform may have had a small but meaningful effect on the average sales of candy. However, the statistical evidence is weak. The average model (Model 2) suggested a reduction of 4.9% in sales (*p*-value = 0.08), but the results of the local model (Model 1) was in the opposite direction. The supplementary analysis of taxed candy with additional control seasons back to 2012 yielded a reduction in sales of 3.9% (p-value = 0.05), and the analysis of candy with snacks as a control product gave a 7.2% reduction (p-value = 0.06). *P*-values in this range are not unexpected given the four main analyses and the twelve supplementary analyses.^1^ Descriptive analyses showed that the price per volume across subcategories of candy and soda increased during the intervention season by respectively 5.8 and 8.0 percentage points beyond the changes in the control season.

The present results partly contrast with some of the past literature on the impact of taxes on unhealthy foods and beverages, especially concerning beverages. Empirical studies from Mexico and the US reveal reductions in sales or purchases of beverages after implementation of taxes on beverages, however, with varying effects [21–24]. Findings from the tax on beverages in Philadelphia in the US, indicated an overall reduction of 38% in sales of taxed beverages, despite large increases in volumes sales in bordering zip codes [24]. Furthermore, sales in Berkeley one-year post implementation of a SSB-tax declined by 9.6%; however, it increased by 6.9% in non-Berkeley stores [25]. Average weekly sales in Barbados decreased with 4.3% following implementation of a SSB-tax, compared to expected sales without a tax [21]. A study using self-scanned purchases from a panel of 6253 Mexican households reported a 6% reduction in taxed beverages after the implementation of a SSB-tax [23]. As an example of more inconclusive results, the evaluation of a French soda tax reported mixed evidence from analyses of purchase responses [26].

In comparison with some of the results on beverages, evaluations of taxes on unhealthy food products show smaller reductions or substitution effects in purchases [12–14, 27]. For example, evaluations of the tax on energy-dense foods in Mexico revealed a decrease in purchases of 5.3% on taxed foods (in 2014–2016) when compared to a period without taxes (2008–2012) [12].

In contrast to our findings, Steen and Ulsaker (2019) reported a 23% reduction in chocolate sales and an 11% reduction in soda sales when evaluating the same increases in the Norwegian tax on a smaller sample from the same data as used in the present study [28].

One difference between several of the studies cited above, and the present one is that we have accounted for dependencies within each time unit by calculating standard errors that are cluster-robust at the dimensions of both time and geography. To illustrate the impact of such dependencies, we ran a model without adjusting for clustering on time, and we obtained a substantial reduction in the estimated uncertainty. However, the inference in that model assumes independence among the observations between the geographical clusters, which means that unmodelled co-movement in the sales at the national or cross-regional level (due to sales promotions, weather, sports events, etc.) produce pseudo-replication. For more details on cluster-robust inference, see Cameron et al. (2006) [29] and Abadie et al. (2017) [30]. Note, however, that several studies use aggregated data for which clustering is not an issue [12, 21].

Taxing unhealthy foods and beverages have become more common, yet it is still difficult to compare effects between countries. The contexts differ in terms of initial intake levels of the taxed products, the level and design of the tax, product market, economy, and cross-border shopping. Furthermore, other uncertainties and differences related to price transmission, consumer response, and substitution patterns make comparisons challenging.

Although the meta-analysis by Teng et al. (2019) reported that the equivalent of a 10% SSB-tax was associated with an average decline in beverage purchases and dietary intake of 10%, they concluded that context and tax design might be just as important as the tax level in designing SSB-taxes for maximum impact [11]. Other studies suggest that for taxes to affect consumption, taxes need to increase prices for consumers with 20% or more [6, 10], which is substantially more than our estimated differences from pre to post tax of 5.8 and 8.0 percentage points for candy and soda respectively.

The increase in the taxes should have made an 80-g chocolate bar increase with about 1.5 NOK (0.14 EUR) which includes the value added tax of 15%, and a 0.5-l soda should have increased with about 0.8 NOK (0.08 EUR). The absolute price of the products varies substantially between periods, different types of products, and brands. The absolute change in price for the taxed products is unknown as the stores set the price on products, whereas the taxes are levied producers of the products. Although we reported the changes in price per volume, we do not have access to details about differences in sales between brands. Therefore, our estimate of the changes in price may not accurately reflect how much of the tax was passed on to the consumers. Furthermore, Norwegians use a small part of their income on foods and nonalcoholic beverages (about 12%) and are considered to have high purchasing power [31]. Altogether, this suggests that the tax level of unhealthy products in Norway needs to be substantially higher for the consumer to affect purchase behavior to a larger extent. Nevertheless, the taxes were mainly increased to create revenues. Thus, the increases in the taxes had the intended effect. Taxes with the aim of improving public health, need to be designed accordingly.

### Strengths and limitations

This study is from a real-world setting, and our data consist of almost all annual sales in Norwegian grocery stores, which we consider as strengths. Additionally, in a study like the present one, where the choices are numerous regarding taxed product groups, comparison products, control periods, length of the intervention period, and statistical modeling, the pre-registration of methods and hypothesis is a major strength. This precludes the possibility that we have tweaked our model to obtain more interesting results or results in line with political, government, or business interests.

The differences in the signs of the coefficients between Model 1 (local effect) and Model 2 (average effect) may be attributed to the high variability in sales over time. As sales vary by weather conditions, marketing campaigns, holidays, etc., we could have obtained higher precision if we had achieved better control of variables that influence sales. The analysis that used data back to 2012 gave a more powerful test, but it also implicated seasons that were more distal to the intervention, and this data may exhibit different seasonal patterns. Furthermore, the actual impact of the tax increases on consumer prices is uncertain. It has been suggested that it can take at least 6 months until taxes are fully passed onto consumer prices [32].

In the present study, we used differences in changes as means to draw conclusions about the causal impact of the tax policy. Although we modeled the pre-existing trends in sales and controlled for a proxy of total sales, we cannot control for unknown factors that selectively affect sales of the taxed product groups in the year of the intervention. This is, however, an inherent limitation of all observational studies of this kind. Furthermore, retail data from small, independent stores with foreign products are not included, nor is data from kiosks and gas stations. As taxed products are sold in these venues, we cannot exclude possible effects of the taxes in these outlets. A limitation concerning the use of retail sales data is that we cannot assess the potential impact on actual consumption for different types of consumers.

## Conclusion

Our results are inconclusive, as we could not consistently detect changes in sales of taxed products after an abrupt increase in taxes on candy and soda. High variation in sales across time resulted in high uncertainty of the effect estimates, which underscores the importance of adjusting standard errors for clustering on the time dimension in policy evaluations. We speculate that the tax increases were too modest to affect the prices to alter sales sufficiently.

## Supplementary information

**Additional file 1: Supplementary Figure S1.** Weeks included in the main analysis, excluding the weeks with high variability sales. **Supplementary Table S2.** Exponentiated regression coefficients [95% CI], main analyses. **Supplementary Text S3.** Categorization of high cross-border municipalities. **Supplementary Text S4.** Analyses excluding high cross-border municipalities. **Supplementary Table S5.** Exponentiated regression coefficients [95% CI], excluding cross-border municipalities. **Supplementary Table S6.** Exponentiated regression coefficients [95% CI], 12-week exclusion around the cutoff. **Supplementary Table S7.** Exponentiated regression coefficients [95% CI], additional control seasons. **Supplementary Table S8.** Exponentiated regression coefficients [95% CI], analyses with control products (difference-in-difference-in-differences).

## Data Availability

The datasets generated and/or analyzed during the current study are proprietary (Nielsen Company Norway).

## Abbreviations

CI: Confidence interval
DiDiDi: Difference-in-difference-in-difference, triple difference design
EUR: Euro
NA: Not applicable
NOK: Norwegian kroner
SD: Standard deviation
SSB: Sugar-sweetened beverages

## Footnotes

1 Although our tests are not independent, the high probability of obtaining at least one *p*-value below 0.05 can be illustrated by assuming a true null and 16 independent tests, 1-(0.95)^16^, which gives a probability of 56%.
