# Supplementary material for: The effects of an abrupt increase in taxes on candy and soda in Norway: an observational study of retail sales
Source: Int J Behav Nutr Phys Act. 2020 Sep 14;17:115. doi: 10.1186/s12966-020-01017-3 (PMC7491168; doi:10.1186/s12966-020-01017-3)
Supplement: Supplementary file 1 — Additional file 1: Supplementary Figure S1. Weeks included in the main analysis, excluding the weeks with high variability sales. Supplementary Table S2. Exponentiated regression coefficients [95% CI], main analyses. Supplementary Text S3. Categorization of high cross-border municipalities. Supplementary Text S4. Analyses excluding high cross-border municipalities. Supplementary Table S5. Exponentiated regression coefficients [95% CI], excluding cross-border municipalities. Supplementary Table S6. Exponentiated regression coefficients [95% CI], 12-week exclusion around the cutoff. Supplementary Table S7. Exponentiated regression coefficients [95% CI], additional control seasons. Supplementary Table S8. Exponentiated regression coefficients [95% CI], analyses with control products (difference-in-difference-in-differences). [file 12966_2020_1017_MOESM1_ESM.zip › 12966_2020_1017_MOESM1_ESM/Additional File 1_ESM.docx]

# Additional File 1

**Supplementary Figure S1:** Weeks included in the main analysis, excluding the weeks with high variability sales.

**Supplementary Table S2**: Exponentiated regression coefficients [95% CI], main analyses.

**Supplementary Text S3**: Categorization of high cross-border municipalities.

**Supplementary Text S4**: Analyses excluding high cross-border municipalities.

**Supplementary Table S5**: Exponentiated regression coefficients [95% CI], excluding cross-border municipalities.

**Supplementary Table S6**: Exponentiated regression coefficients [95% CI], 12-week exclusion around the cutoff.

**Supplementary Table S7**: Exponentiated regression coefficients [95% CI], additional control seasons.

**Supplementary Table S8**: Exponentiated regression coefficients [95% CI], analyses with control products (difference-in-difference-in-differences).

## Supplementary Figure S1:

**Figure S1.** Weeks included in the main analysis, excluding the weeks with high variability sales.

Pre/post signifies pre or post the cut-off (January 1^st^). Tax increases were implemented from January 1^st^ 2018.

## Supplementary Table S2 - Main analyses

| **Table S2. Exponentiated regression coefficients [95% CI], main analyses.** | | | | |
| --- | --- | --- | --- | --- |
|  | **Candy** | | **Soda** | |
|  | Model 1 (local) | Model 2 (average) | Model 1 (local) | Model 2 (average) |
| Tax effect | 1.061 | 0.951 | 0.961 | 1.015 |
|  | [NA, 1.234] | [NA, 1.010] | [NA, 1.049] | [NA, 1.050] |
| Nonedible products | 1.867*** | 1.865*** | 1.757*** | 1.768*** |
|  | [1.731, 2.013] | [1.735, 2.005] | [1.601, 1.929] | [1.612, 1.941] |
| Easter |  | 1.456*** |  | 1.236*** |
|  |  | [1.256, 1.689] |  | [1.160, 1.317] |
| Easter2017 | 1.397*** |  | 1.218*** |  |
|  | [1.310, 1.490] |  | [1.159, 1.279] |  |
| Easter2018 | 1.329*** |  | 1.169*** |  |
|  | [1.232, 1.432] |  | [1.105, 1.236] |  |
| Halloween2016 | 0.970* |  | 0.992 |  |
|  | [0.942, 0.998] |  | [0.964, 1.022] |  |
| Halloween2017 | 1.061 |  | 0.992 |  |
|  | [0.994, 1.132] |  | [0.962, 1.023] |  |
| Dur_cubic1 | 1.011*** |  | 1.002 |  |
|  | [1.006, 1.016] |  | [0.996, 1.008] |  |
| Dur_cubic2 | 0.986*** |  | 1.004 |  |
|  | [0.979, 0.992] |  | [0.997, 1.012] |  |
| Dur_cubic1_t | 1.007 |  | 1.002 |  |
|  | [0.998, 1.016] |  | [0.995, 1.009] |  |
| Dur_cubic2_t | 0.984** |  | 1.002 |  |
|  | [0.973, 0.996] |  | [0.992, 1.012] |  |
|  |  |  |  |  |
| Shop FE | Yes | Yes | Yes | Yes |
| Week FE | No | Yes | No | Yes |
| Number of time clusters | 76 | 76 | 76 | 76 |
| Number of municipality clusters | 428 | 428 | 428 | 428 |
| Number of shops | 3884 | 3884 | 3884 | 3884 |
| Number of observations | 278977 | 278977 | 278982 | 278982 |
| * p <0.05, ** p <0.01, *** p <0.001. CI: Confidence interval. Dur_cubic1/Dur_cubic2: restricted cubic splines, shared time trends. Dur_cubic2_t/Dur_cubic2_t: deviation from time trend in intervention season. FE: Fixed effect. NA: Not applicable due to one-sided Ci. | | | | |

## Supplementary Text S3 - Categorization of high cross-border municipalities

We categorized municipalities as high cross-border in two steps: (1) Meat is one of the products mainly bought across the border^[[1]](#footnote-1)^; thus, bacon was used as a meat indicator as this product is marketed as a border-shopping product. We calculated bacon sales before the tax increases (with 2017 data) as weekly proportions of all weekly sales (in NOK) within each store, and flagged municipalities where the proportion was under the 25-percentile (<0.9%). (2) From the municipalities flagged with low bacon sales, we categorized municipalities with easy access to cross-border shopping areas (by ferry or car). Easy access by car was estimated as <90 minutes driving distance from one of the four main cross-border areas or <60 minutes from a main ferry harbor to Denmark.

## Supplementary Text S4 – Analyses excluding high cross-border municipalities

Of the 107 municipalities with the lowest bacon sales, 47 municipalities had a <90-minute drive to one of the four major cross-border shopping areas and 16 municipalities had <60 minutes driving distance to a major ferry terminal. Thus, 63 municipalities (of 428) were categorized as high cross-border municipalities. Of 3884 individual shops, 1098 were removed, thus in the analyses without high cross-border municipalities, 2786 unique shops were included. The results are shown in Supplementary Table S5.

## Supplementary Table S5 – Analyses excluding high cross-border municipalities

| **Table S5. Exponentiated regression coefficients [95% CI], excluding cross-border municipalities.** | | | | |
| --- | --- | --- | --- | --- |
|  | **Candy** | | **Soda** | |
|  | Model 1 (local) | Model 2 (average) | Model 1 (local) | Model 2 (average) |
| Tax effect | 1.054 | 0.967 | 0.970 | 1.015 |
|  | [NA, 1.229] | [NA, 1.026] | [NA, 1.061] | [NA, 1.051] |
| Nonedible products | 1.869*** | 1.859*** | 1.754*** | 1.757*** |
|  | [1.731, 2.019] | [1.727, 2.001] | [1.593, 1.930] | [1.598, 1.931] |
| Easter |  | 1.513*** |  | 1.309*** |
|  |  | [1.320, 1.734] |  | [1.246, 1.375] |
| Easter2017 | 1.428*** |  | 1.261*** |  |
|  | [1.339, 1.524] |  | [1.201, 1.325] |  |
| Easter2018 | 1.379*** |  | 1.235*** |  |
|  | [1.278, 1.488] |  | [1.166, 1.307] |  |
| Halloween2016 | 0.961** |  | 0.989 |  |
|  | [0.933, 0.990] |  | [0.959, 1.020] |  |
| Halloween2017 | 1.052 |  | 0.987 |  |
|  | [0.985, 1.124] |  | [0.957, 1.018] |  |
| Dur_cubic1 | 1.009*** |  | 1.001 |  |
|  | [1.004, 1.014] |  | [0.994, 1.007] |  |
| Dur_cubic2 | 0.987*** |  | 1.006 |  |
|  | [0.980, 0.994] |  | [0.998, 1.014] |  |
| Dur_cubic1_t | 1.006 |  | 1.002 |  |
|  | [0.997, 1.016] |  | [0.994, 1.009] |  |
| Dur_cubic2_t | 0.986* |  | 1.001 |  |
|  | [0.975, 0.998] |  | [0.991, 1.011] |  |
|  |  |  |  |  |
| Shop FE | Yes | Yes | Yes | Yes |
| Week FE | No | Yes | No | Yes |
| Number of time clusters | 76 | 76 | 76 | 76 |
| Number of municipality clusters | 365 | 365 | 365 | 365 |
| Number of shops | 2786 | 2786 | 2786 | 2786 |
| Number of observations | 201461 | 201461 | 201466 | 201466 |
| * p <0.05, ** p <0.01, *** p <0.001. CI: Confidence interval. Dur_cubic1/Dur_cubic2: Restricted cubic splines, shared time trends. Dur_cubic2_t/Dur_cubic2_t: Deviation from time trend in intervention season. FE: Fixed effect. NA: Not applicable due to one-sided CI. | | | | |

## Supplementary Table S6 – Analyses with an additional number of weeks excluded

| **Table S6. Exponentiated regression coefficients [95% CI], 12-week exclusion around the cutoff.** | | | | |
| --- | --- | --- | --- | --- |
|  | **Candy** | | **Soda** | |
|  | Model 1 (local) | Model 2 (average) | Model 1 (local) | Model 2 (average) |
| Tax effect | 1.159 | 0.961 | 0.965 | 1.019 |
|  | [NA, 1.323] | [NA, 1.027] | [NA, 1.071] | [NA, 1.058] |
| Nonedible products | 1.875*** | 1.879*** | 1.769*** | 1.780*** |
|  | [1.736, 2.026] | [1.745, 2.023] | [1.610, 1.945] | [1.620, 1.956] |
| Easter |  | 1.457*** |  | 1.237*** |
|  |  | [1.260, 1.686] |  | [1.160, 1.318] |
| Easter2017 | 1.379*** |  | 1.219*** |  |
|  | [1.287, 1.479] |  | [1.158, 1.282] |  |
| Easter2018 | 1.278*** |  | 1.175*** |  |
|  | [1.178, 1.386] |  | [1.100, 1.255] |  |
| Halloween2016 | 0.967* |  | 1.010 |  |
|  | [0.935, 0.999] |  | [0.976, 1.045] |  |
| Halloween2017 | 1.110*** |  | 1.013 |  |
|  | [1.057, 1.166] |  | [0.981, 1.045] |  |
| Dur_cubic1 | 1.014*** |  | 0.999 |  |
|  | [1.008, 1.020] |  | [0.992, 1.006] |  |
| Dur_cubic2 | 0.981*** |  | 1.006 |  |
|  | [0.972, 0.989] |  | [0.997, 1.016] |  |
| Dur_cubic1_t | 1.003 |  | 1.001 |  |
|  | [0.994, 1.012] |  | [0.993, 1.009] |  |
| Dur_cubic2_t | 0.983* |  | 1.003 |  |
|  | [0.970, 0.997] |  | [0.991, 1.016] |  |
|  |  |  |  |  |
| Shop FE | Yes | Yes | Yes | Yes |
| Week FE | No | Yes | No | Yes |
| Number of time clusters | 68 | 68 | 68 | 68 |
| Number of municipality clusters | 428 | 428 | 428 | 428 |
| Number of shops | 3883 | 3883 | 3883 | 3883 |
| Number of observations | 249585 | 249585 | 249589 | 249589 |
| * p <0.05, ** p <0.01, *** p <0.001. CI: Confidence interval. Dur_cubic1/Dur_cubic2: Restricted cubic splines, shared time trends. Dur_cubic2_t/Dur_cubic2_t: Deviation from time trend in intervention season. FE: Fixed effect. NA: Not applicable due to one-sided CI. | | | | |

## Supplementary Table S7 – Model 2 with additional control seasons

| **Table S7. Exponentiated regression coefficients [95% CI], additional control seasons.** | | |
| --- | --- | --- |
|  | **Candy** | **Soda** |
|  | Model 2 (average) | Model 2 (average) |
| Tax effect | 0.951* | 1.055 |
|  | [NA, 0.999] | [NA, 1.093] |
| Nonedible products | 1.991*** | 1.883*** |
|  | [1.918, 2.068] | [1.804, 1.965] |
| Easter | 1.386*** | 1.268*** |
|  | [1.203, 1.598] | [1.190, 1.350] |
| Intervention season | 1 (reference) | 1 (reference) |
|  | [1, 1] | [1, 1] |
| Season 2016-2017 | 0.944** | 0.977 |
|  | [0.911, 0.977] | [0.948, 1.006] |
| Season 2015-2016 | 0.940** | 0.978 |
|  | [0.904, 0.977] | [0.945, 1.011] |
| Season 2014-2015 | 0.995 | 1.000 |
|  | [0.960, 1.031] | [0.968, 1.032] |
| Season 2013-2014 | 0.966 | 0.991 |
|  | [0.930, 1.004] | [0.961, 1.021] |
| Season 2012-2013 | 1.002 | 1.005 |
|  | [0.968, 1.037] | [0.976, 1.034] |
|  |  |  |
| Shop FE | Yes | Yes |
| Week FE | Yes | Yes |
| Number of time clusters | 228 | 228 |
| Number of municipality clusters | 430 | 430 |
| Number of shops | 4797 | 4797 |
| Number of observations | 836841 | 836845 |
| * p <0.05, ** p <0.01, *** p <0.001. CI: Confidence interval. FE: Fixed effect. NA: Not applicable due to one-sided CI. | | |

## Supplementary Table S8 – Model 2 with control products snacks and water

| **Table S8. Exponentiated regression coefficients [95% CI], analyses with control products (difference-in-difference-in-differences).** | | |
| --- | --- | --- |
|  | **Candy vs. snacks** | **Soda vs. water** |
|  | Model 2 (average) | Model 2 (average) |
| Tax effect | 0.928 | 1.008 |
|  | [NA, 1.005] | [NA, 1.173] |
| Intervention season | 1.049*** | 1.058 |
|  | [1.020, 1.079] | [0.974, 1.150] |
| Intervention season by cutoff | 1.010 [0.961, 1.063] | 1.043 [0.929, 1.171] |
| Intervention season by product category | 0.971  [0.915, 1.030] | 0.927 [0.791, 1.087] |
| Easter | 1.316*** | 1.051 |
|  | [1.194, 1.452] | [0.978, 1.129] |
|  |  |  |
| Week FE | Yes | Yes |
| Shop by product category FE | Yes | Yes |
|  |  |  |
| Number of time clusters | 76 | 76 |
| Number of municipality clusters | 428 | 428 |
| Number of shops | 7769 | 7767 |
| Number of observations | 557977 | 554742 |
| * p <0.05, ** p <0.01, *** p <0.001. CI: Confidence interval. FE: Fixed effect. NA: Not applicable due to one-sided CI. The effect of product category is absorbed in Shop by product category FE. Tax Effect = three-way interaction between Intervention season, cutoff, and product category. | | |

1. Steen, F., Friberg, R., Ulsaker, S., *Hump-shaped Cross-price Effects and the Extensive Margin in Cross-border Shopping.* NHH Dept. of Economics Discussion Paper., 2018. [↑](#footnote-ref-1)
