# Supplementary figures and images for: The effects of an abrupt increase in taxes on candy and soda in Norway: an observational study of retail sales
Source: Int J Behav Nutr Phys Act. 2020 Sep 14;17:115. doi: 10.1186/s12966-020-01017-3 (PMC7491168; doi:10.1186/s12966-020-01017-3)

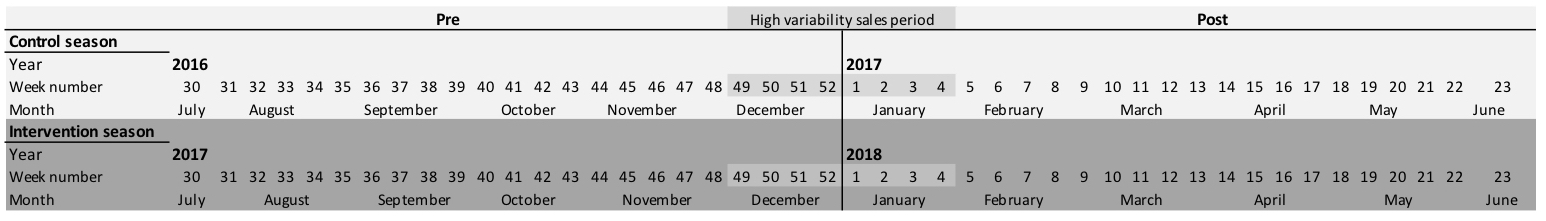

Supplement: Supplementary file 1 — Additional file 1: Supplementary Figure S1. Weeks included in the main analysis, excluding the weeks with high variability sales. Supplementary Table S2. Exponentiated regression coefficients [95% CI], main analyses. Supplementary Text S3. Categorization of high cross-border municipalities. Supplementary Text S4. Analyses excluding high cross-border municipalities. Supplementary Table S5. Exponentiated regression coefficients [95% CI], excluding cross-border municipalities. Supplementary Table S6. Exponentiated regression coefficients [95% CI], 12-week exclusion around the cutoff. Supplementary Table S7. Exponentiated regression coefficients [95% CI], additional control seasons. Supplementary Table S8. Exponentiated regression coefficients [95% CI], analyses with control products (difference-in-difference-in-differences). [file 12966_2020_1017_MOESM1_ESM.zip › 12966_2020_1017_MOESM1_ESM/Figure S1.jpg]
